# Diminished growth and vitality in juvenile *Hydractinia echinata* under anticipated future temperature and variable nutrient conditions

**DOI:** 10.1038/s41598-021-86918-4

**Published:** 2021-04-05

**Authors:** Daniel Tschink, Gabriele Gerlach, Michael Winklhofer, Cora Kohlmeier, Bernd Blasius, Laura Eickelmann, Yvonne Schadewell, Julia Strahl

**Affiliations:** 1 Institute of Biology and Environmental Sciences, Carl Von Ossietzky University Oldenburg, Carl von Ossietzky Str. 9-11, 26111 Oldenburg, Germany; 2 Institute for Chemistry and Biology of the Marine Environment (ICBM), Carl Von Ossietzky University Oldenburg, Carl von Ossietzky Str. 9-11, 26111 Oldenburg, Germany; 3 Helmholtz Institute for Functional Marine Biodiversity at the University of Oldenburg (HIFMB), Ammerländer Heerstr. 231, 26129 Oldenburg, Germany; 4 Centre of Excellence for Coral Reef Studies and School of Marine and Tropical Biology, James Cook University, Townsville, QLD 4811 Australia; 5 Alfred Wegener Institute Helmholtz Centre for Polar and Marine Research, Am Handelshafen 12, 27570 Bremerhaven, Germany

**Keywords:** Climate-change ecology, Ecophysiology

## Abstract

In a warming climate, rising seawater temperatures and declining primary and secondary production will drastically affect growth and fitness of marine invertebrates in the northern Atlantic Ocean. To study the ecological performance of juvenile hydroids *Hydractinia echinata* we exposed them to current and predicted water temperatures which reflect the conditions in the inter- and subtidal in combination with changing food availability (high and low) in laboratory experiments. Here we show, that the interplay between temperature stress and diminished nutrition affected growth and vitality of juvenile hydroids more than either factor alone, while high food availability mitigated their stress responses. Our numerical growth model indicated that the growth of juvenile hydroids at temperatures beyond their optimum is a saturation function of energy availability. We demonstrated that the combined effects of environmental stressors should be taken into consideration when evaluating consequences of climate change. Interactive effects of ocean warming, decreasing resource availability and increasing organismal energy demand may have major impacts on biodiversity and ecosystem function.

## Introduction

Worldwide, marine ecosystems are threatened by the impacts of climate change^1^, and there is intense debate as to whether acclimation or adaption in marine organisms will keep pace with the rate of ecosystem changes predicted for the future^2^. Rising sea surface temperature (SST) and shifts in biogeochemical cycles of the world´s oceans, and therewith in food availability, affects the ecological performance and fitness of marine biota^3^. There is good evidence that ocean warming can drive species’ turnover^4^ and species-specific changes in phenology and trophic mismatches^5^, leading to community and ecosystem shifts.

Global SST already increased by 0.11 °C per decade between 1970 and 2010^6^, and are predicted to further increase by 1.5–2 °C by 2100^7^. Due to topographic conditions (e.g. shallow depths, high tidal ranges, large scale intertidal zones) SST in shelf seas such as the North Sea are expected to increase by 3°C^8^. Increasing frequencies of extreme weather events, such as storms^9^, and more intense and longer-lasting heat waves^10^ might intensify temperature stress, especially in exposed habitats such as the intertidal^11^.

Within the next few decades, ocean warming is predicted to cause a decrease in primary and secondary production in the northern Atlantic Ocean^12^. This, in combination with changing global climate patterns, such as the North Atlantic Oscillation^13^, and in local ocean dynamics (e.g. wave action, storm events)^14,15^, might limit food availability, feeding time, and prey-capture success of marine benthic invertebrates in the future^16^. In addition, rising SST may shift the seasonal occurrence and abundance of primary and secondary producers, which may induce trophic mismatches and reduce the ecological performance of higher trophic organisms^17^.

While the effects of changing water temperatures on benthic and pelagic ectotherms have been investigated in recent decades (e.g.^18–20^), temperature-nutrient interactions in temperate marine ectotherms have received little attention. Their ecological consequences are difficult to assess, due to the complexity of the organism—environment interactions and of the interacting physiological and ecological processes underlying stress resistance, resilience and acclimation/adaptation of marine organisms to predicted environmental changes^21^. It is therefore essential to understand how potential environmental stresses (e.g. SST beyond the optimal range) may affect their vitality^22^. Ectotherms, for instance, are less tolerant to warming than to cooling^23^, and there is substantial evidence that rising temperatures affect physiological fitness, increase costs for somatic maintenance, and alter growth and the competitive potential^22–24^. Especially in the intertidal, temperature fluctuations caused by tides may increase temperature stress in the future and potentially intensify the effects of temperature-nutrient interactions on marine organisms.

The marine hydroid *Hydractinia echinata* (Flemming, 1828) is a suitable model organism, as it is widely distributed in the Northern Atlantic Ocean^25^ and characterized by high morphological plasticity^26^. Its distribution pattern indicates a high tolerance to a broad range of environmental conditions (e.g., different temperature and salinity ranges). Thus the hydroids can be found in the intertidal in < 1 m water depth, as well as in the subtidal and even beyond > 50 m depth at different locations in the North Atlantic Ocean^27^. Juvenile colonies consist exclusively of feeding polyps (Gastrozooids) interconnected by a stolon system^26^, which take up and decompose food. The resulting energy resources are distributed through the stolon system and serve the somatic maintenance and growth of the young animals.

To test whether the high morphological plasticity and high tolerances of *H. echinata* to variable environmental conditions are corresponding to a high acclimation potential of juvenile hydroids to predicted environmental conditions in the North Sea, we ran two controlled aquarium experiments. In the first experiment (scenario 1), treatments correlated to temperature conditions in subtidal habitats of the German Bight in summer, while the second experiment (scenario 2) simulated temperature fluctuations in intertidal habitats. In both experiments, two temperature regimes (ambient and high) were cross-factored with two food regimes (high and low) to study the growth performance (polyp number, colony area) and mortality rates of young animals under eight different experimental conditions. We then developed a numerical growth model based on the assumptions of a temperature-dependent metabolism and the amount of available energy to identify general stress-response mechanisms, to compare experimental with model data. Our research investigated three main topics: (1) The influence of rising summer SST and varying food conditions on morphology and mortality rates of juvenile *H. echinata*; (2) The effect of fluctuating temperature stresses, such as those found in the intertidal, on growth performance; and (3) The identification of possible stress-response mechanisms in juvenile hydroids in different habitats by comparing experimental data with model simulations.

## Results

### Growth rates and mortality

**Figure 1:**
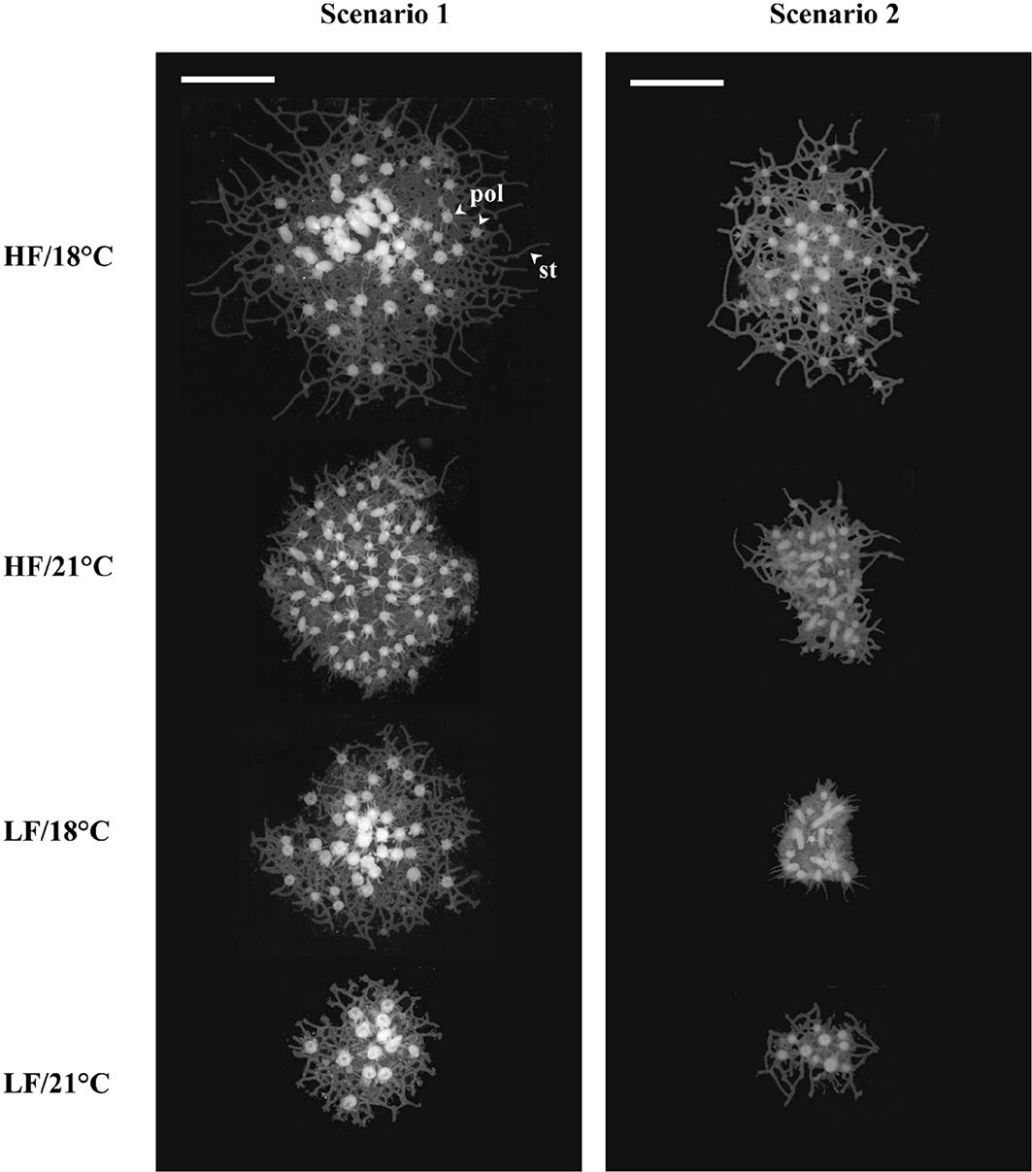
Representative images of juvenile *H. echinata* colonies in all treatments of scenario 1(left) and 2 (right) by the end of both experiments. HF = high food, LF = low food, 18 °C = control temperature, 21 °C = high temperature, st = stolon channels, pol = polyps. The white scale bar equals 2 mm.

**Figure 2:**
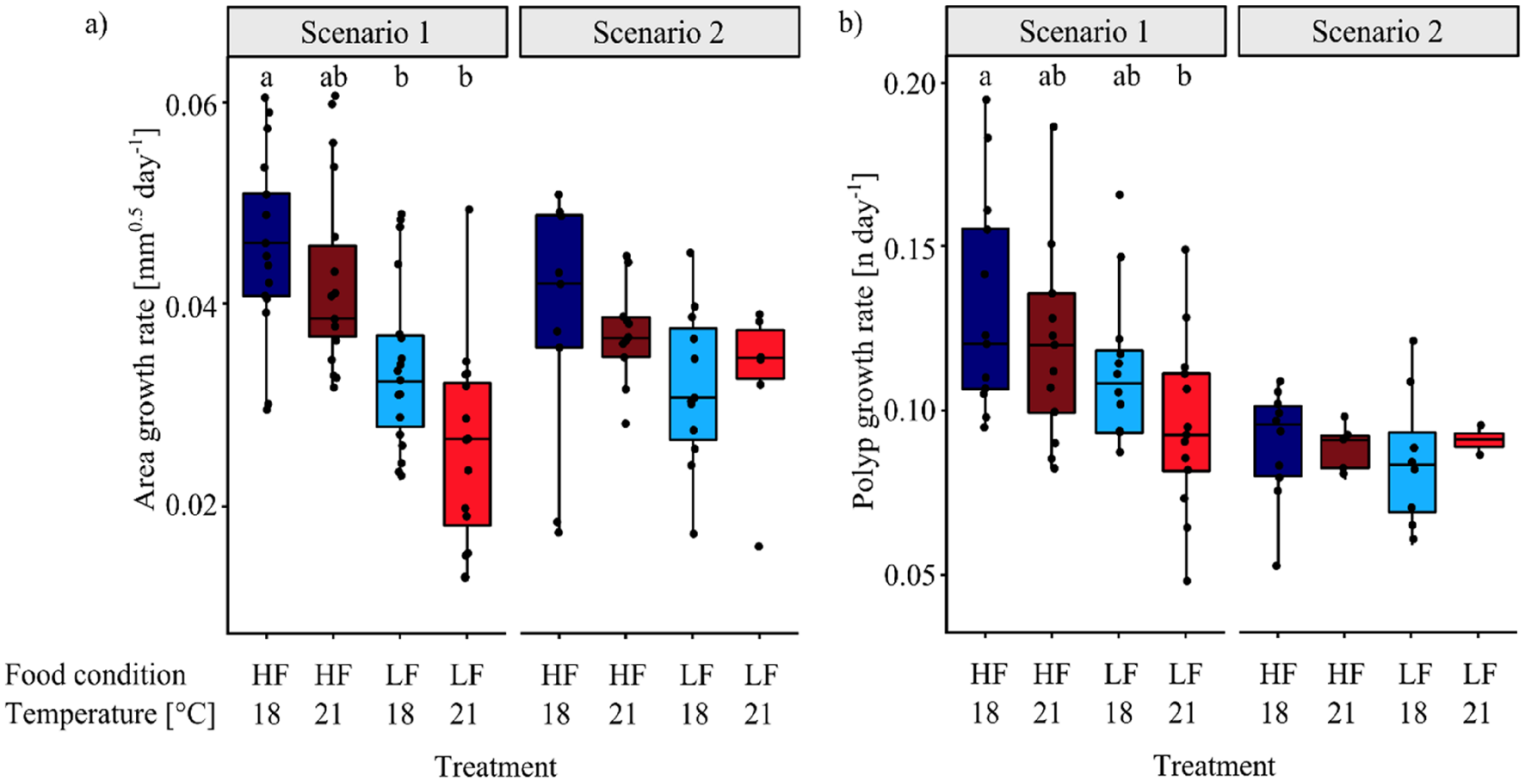
Area growth rates (**a**) and polyp growth rates (**b**) of juvenile *H. echinata* in two experiments, scenario 1(left) and 2 (right); median ± SD . HF = high food (dark blue and red boxes), LF = low food (light blue and red boxes), 18 °C = control temperature (blue boxes), 21 °C = high temperature (red boxes). All treatments in scenario 2 contained an extra temperature step of + 1.5 °C for six hours daily (see also Fig. 3). Significant differences between treatments are marked by lower cases.

In both scenarios (scenario 1 = subtidal; scenario 2 = intertidal), growth rates of juvenile *H. echinata* were affected by food and temperature. Typically, colony area and polyp number were lower at the higher temperature (21 °C) combined with insufficient food availability (low food, LF) (Figs. 1, 2; Supplementary Tables 2 and 3). Interestingly, growth performance was generally more affected by food limitation than by temperature stress.

In scenario 1, the juvenile growth rates were highest at HF/18 °C and lowest at LF/21 °C (Fig. 2), with a reduction by 77% in area growth rate (*p* < 0.001, Table 1, Fig. 2a) and by 72% in polyp growth rate (*p* = 0.013, Table 1, Fig. 2b) (Supplementary Tables 2 and 3). In contrast, no significant differences in area and polyp growth rates were detected between HF/18 °C and HF/21 °C. Low food availability alone (LF/18 °C) significantly decelerated colony area growth by 50% (*p* = 0.001, Table 1) compared to HF/18 °C, but had a less pronounced effect on polyp number growth (decrease by 32%, n.s.). The effect of food scarcity was also visible in the high-temperature treatments, where colonies at LF/21 °C were 66% smaller and developed 63% fewer polyps compared to HF/21 °C (not significant due to high scatter and mortality in the LF/21 °C treatment).

**Table 1:** Statistical results of growth and mortality rates of juvenile *H. echinata* over time in scenario 1 and 2. HF = high food, LF = low food, 18 °C = control temperature, 21 °C = high temperature. Significant values are shaded grey. The upper table contains the *p*-values (*p*), adjusted p-values (*p* adj.) and coefficient of determination (*R*^2^) of pairwise comparison (Wilcoxon test) with multiple-testing adjustment (Bonferroni-Holmes) of area and polyp growth. The bottom part of the table shows Chi-squared (*X*^2^), degrees of freedom (*D*_*f*_) and p-values (*p*) of the test for equality of proportions of the mortality rates. N_Total_ = 77 for scenario 1and 40 for scenario 2.

	Scenario 1						Scenario 2					
	Area			Polyps			Area			Polyps		
**Growth rates**												
*R*^2^	0.90			0.98			0.90			0.98		
	*p*	*p* adj		*p*		*p* adj	*p*	*p* adj		*p*		*p* adj
HF/18 °C–HF/21 °C	0.13	0.13		0.42		0.42	0.49	0.49		0.59		1
HF/18 °C–LF/18 °C	5.3*10^–4^	1.1*10^–3^		0.10		0.2	0.13	0.39		0.63		1
HF/18 °C–LF/21 °C	3.6*10^–6^	1.1*10^–5^		4.2*10^–3^		0.013	0.14	0.39		0.91		1
**Mortality rates**												
	*X*^2^		*D*_*f*_		*p*		*X*^2^		*D*_*f*_		*P*	
	0.22		3		0.97		16.42		3		< 0.001	

Despite a decreasing trend in hydroid growth (i.e., number of polyps, Fig. 2b) with increasing experimental stress in scenario 2, differences between the treatments were statistically not significant. This may be due to high mortality rates in the LF/21 °C, resulting in a lower number of available data points. Under theses high food and temperature stresses (LF/21 °C), the colonies were often not able to develop new polyps for several days to weeks. These “stable” polyp numbers under stressful conditions (LF/21 °C), could not be fitted by the Gompertz function and are therefore not represented in the growth rates. Nevertheless, at 18 °C, low food availability (LF/18 °C) caused 46% smaller colonies with 57% fewer polyps compared to high food conditions (HF/18 °C). At elevated water temperature, colonies were 23% smaller, with 23% fewer polyps for low food (LF/21 °C) compared to high food (HF/21 °C).

**Figure 3:**
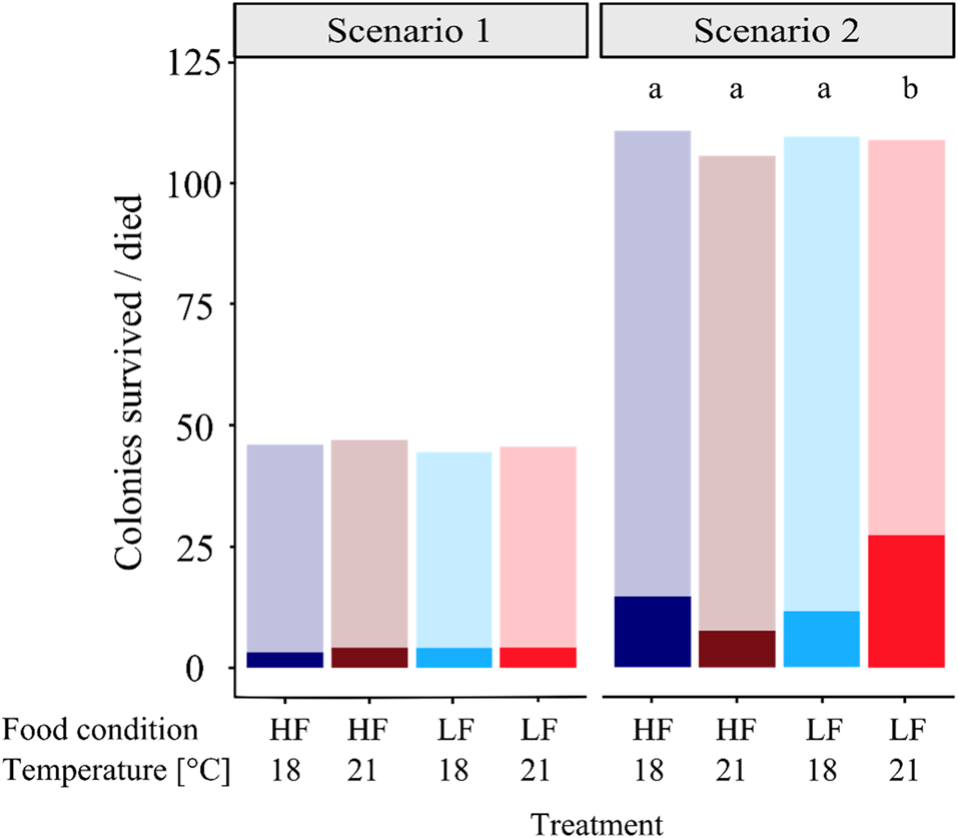
Mortality (full coloured boxes) of juvenile *H. echinata* and surviving colonies (half-transparent boxes) in two experiments, scenario 1(left) and 2 (right); median ± SD. HF = high food (dark blue and red boxes); LF = low food (light blue and red boxes); 18 °C = control temperature (blue boxes); 21 °C = high temperature (red boxes). All treatments in scenario 2 contained an extra temperature step of + 1.5 °C for six hours daily (see also Fig. 3). Significant differences between treatments are marked by lower cases.

The mortality of juveniles in scenario 1 (Fig. 3) did not differ significantly between treatments, ranging between 6.5 and 8.9%. In contrast, the mortality in scenario 2 (Fig. 3) was more heterogeneous and varied between 7.5% in the HF/21 °C treatment and 25.7% in the LF/21 °C. The higher temperature fluctuations in scenario 2 significantly increased mortality of hydroids exposed to the most stressful treatment LF/21 °C (four-sample test of equal proportions, *p* < 0.001). Here, the mortality was almost three times higher compared to all other treatments of both scenarios (Fig. 3).

### Comparison of the growth model and scenario 1

**Figure 4:**
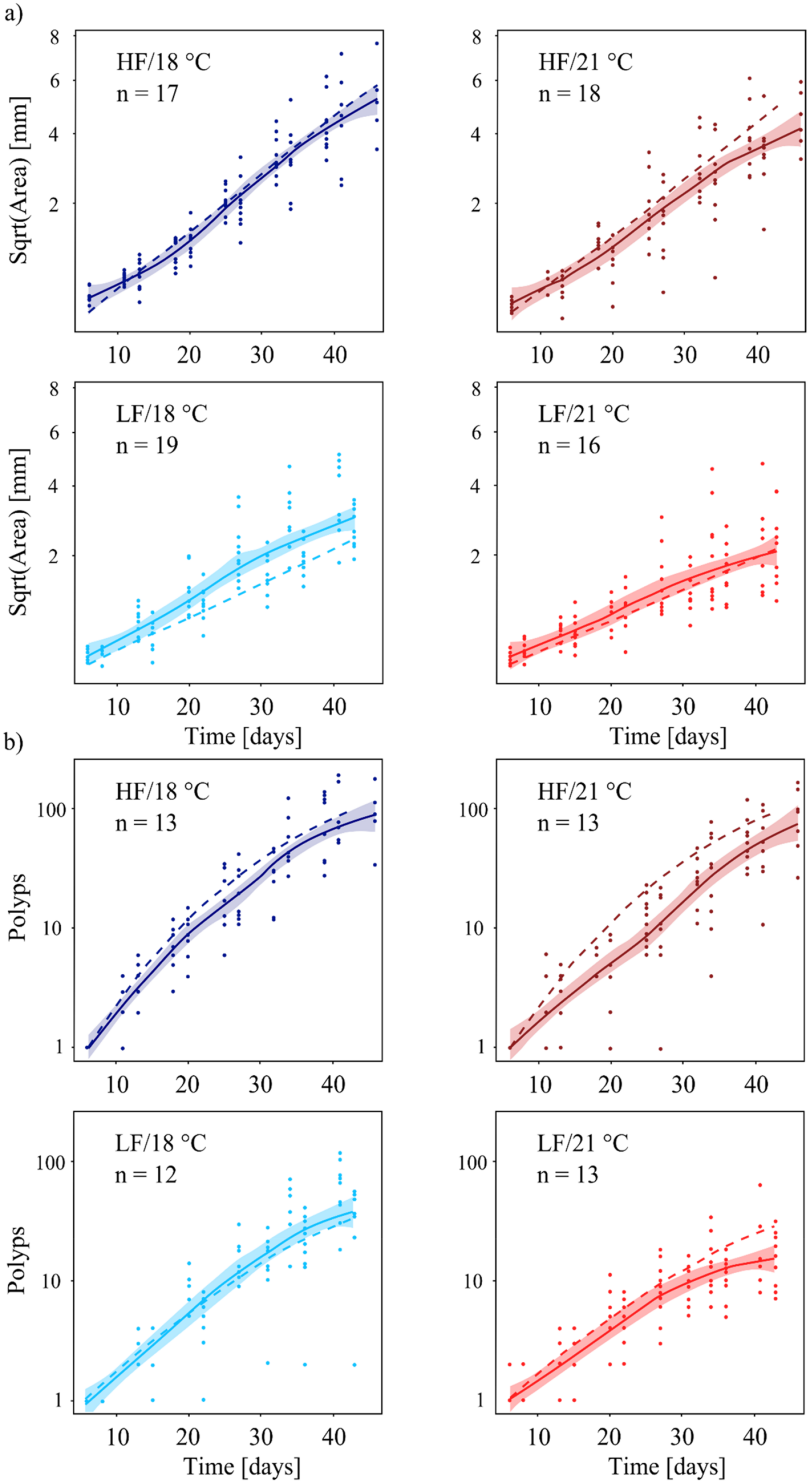
Area (**a**) and polyp number (**b**) of juvenile *H. echinata* in scenario 1over a period of six weeks post-settlement (median ± SE; solid line and shaded region) compared to numerical growth model data (dashed lines). HF = high food (dark blue and red lines); LF = low food (light blue and red lines); 18 °C = control temperature (blue lines); 21 °C = high temperature (red lines).

The area growth (Fig. 4a) in the experimental and simulated data showed a positive, almost linear correlation over time in all treatments. In both data sets, the area of the colonies was affected by food and temperature, resulting in decreased growth under low-food and high-temperature conditions (HF/18 °C < HF/21 °C < LF/18 °C < LF21 °C). Discrepancies between experimental and model data were found in treatments HF/21 °C and LF/18 °C. In contrast to the experiment, that showed lower area growth at elevated temperature (Fig. 4a, HF/21 °C), the model simulated nearly constant growth by the end of the experiment. Furthermore, the model underestimated area growth of the colonies at LF/18 °C (Fig. 4a, bottom left) compared to the experiment, which showed increasing growth rates 20–25 days post settlement. This resulted in larger colonies at the end of the experiment than predicted by the model.

Polyp development (Fig. 4b) over time was more complex than area growth, when comparing experimental with model data. In the high food treatment (Fig. 4b, HF/21 °C), experimental colonies reached the final polyp numbers predicted by the model, but showed a growth delay during the first weeks of the experiment compared to model data. Furthermore, food limitation resulted in lower polyp numbers by the end of the experiment (LF/18 °C, LF/21 °C, Supplementary Table 3) than predicted by the model. In contrast to model predictions, higher water temperatures, in combination with food stress (Fig. 4b, LF/21 °C), limited polyp development towards the end of the experiment. This limitation is reflected by decreasing growth rates at the end of the experiment, and implies that the experimental data are close to saturation compared to the model data.

### Comparison of the growth model and scenario 2

**Figure 5:**
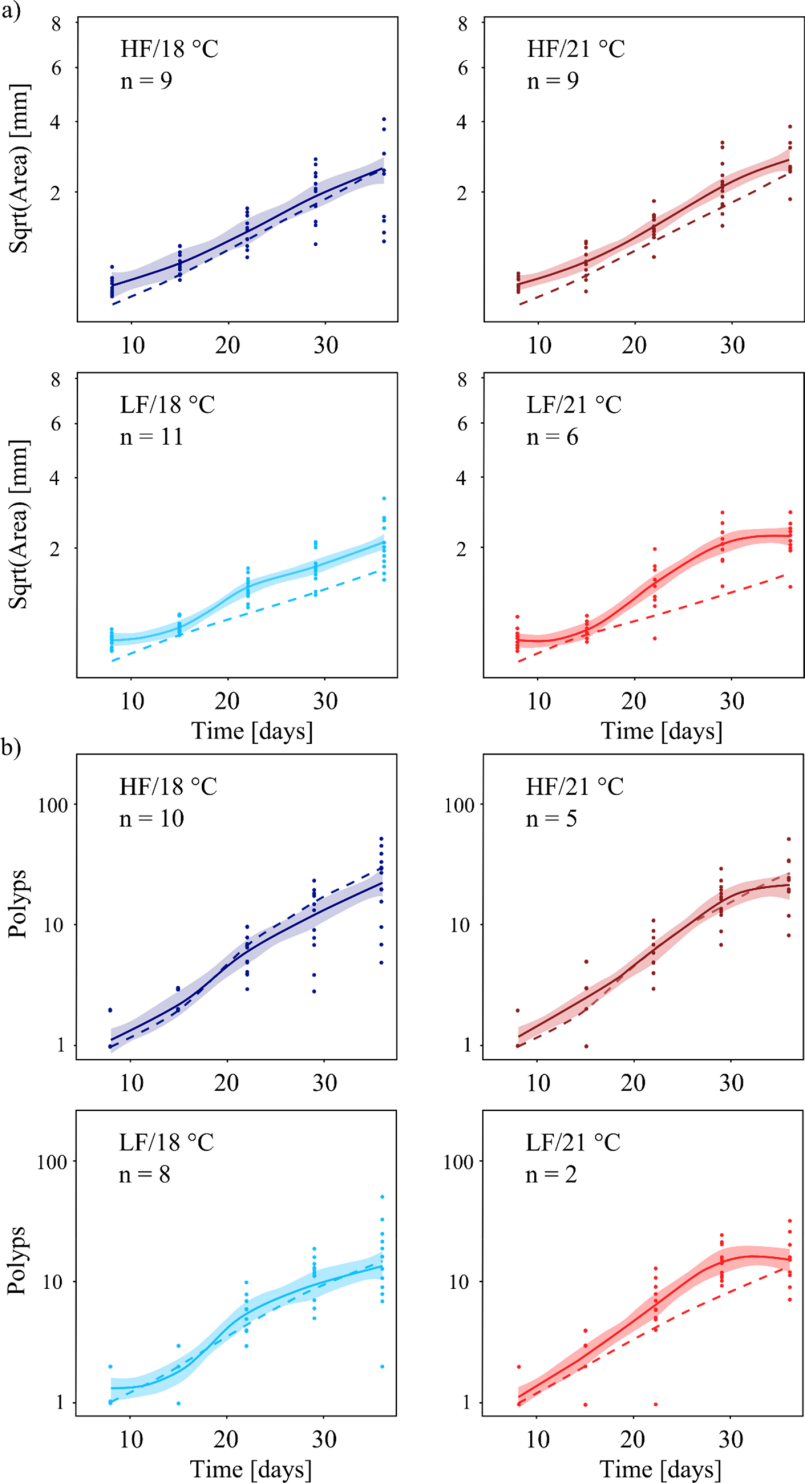
Area (**a**) and polyp number (**b**) of *H. echinata* juveniles in scenario 2 over a period of five weeks post-settlement (median ± SE; solid line and shaded region) compared to numerical growth model data (dashed lines). HF = high food (dark blue and red lines); LF = low food (light blue and red lines); 18 °C = control temperature (blue line); 21 °C = high-temperature (red lines). All treatments contained an extra temperature step of + 1.5 °C for six hours daily (see also Fig. 3).

In scenario 2, temperature fluctuations diminished differences in colony size (= area) between low- and high water temperature in the experimental data (Fig. 5a; Supplementary Table 2). Furthermore, the growth curves based on the experimental data were compressed towards more stressful conditions (stress intensity = HF/18 °C < HF/21 °C < LF/18 °C < LF21 °C), causing an abrupt rise in the growth performance in the low food treatments at about 20 days post-settlement. Saturation of area growth was reached earlier in all experimental treatments compared to HF/18 °C. In most treatments, the area growth was underestimated by the simulation compared to experimental data (HF/21 °C, LF/18 °C, LF/21 °C).

Trends in polyp development (Fig. 5b) in experimental treatments of scenario 2 were similar to scenario 1. Higher water temperature limited growth performance in the experimental data more than predicted by the model. Especially during the last 10 days of the experiment, polyp growth reached saturation, in contrast to the model (Fig. 5b, right, HF/21 °C, LF/21 °C). The experimental polyp number in the most stressful treatment (Fig. 5b, bottom right, LF/21 °C) reached saturation about 30 days post-settlement. The polyp development under these conditions was constantly underestimated by the simulation.

## Discussion

In the present study, low nutrient availability, in combination with temperature stress, considerably reduced vitality and growth performance of *H. echinata*. In scenario 1, mimicking ambient and future environmental conditions in the subtidal (= stable temperature conditions), hydroids showed significantly decreasing polyp numbers and colony area under 21 °C and low food conditions. At the same time, sufficient nutrient availability seemed to mitigate the negative effects of temperature stress on *H. echinata*, leading to similar growth rates of animals exposed to LF/18 °C/and HF/21 °C in both scenarios. The results of scenario 2 further indicate that in the intertidal (= fluctuating temperature conditions), due to high and low tide, higher temperatures will intensify the effects of predicted higher sea water temperatures on marine biota. The additional temperature step of + 1.5 °C (6 h per day) lowered growth rates and diminished differences between the treatments of scenario 2 compared to animals exposed to the more stable temperature conditions in scenario 1. The combination of heat and food stress (LF/21 °C) in scenario 2 actually significantly increased the mortality rate of the hydroids. Our results are supported by studies on marine mollusks, such as bivalves or gastropods^18,28,29^. For example, *Mytilus californianus* responded to the combined effect of heat and food stress with an energetic trade-off between survival and growth^29^. At the lowest trophic level, Thomas and colleagues^22^ demonstrated that temperature-nutrient interactions predicted for 2100 are likely to limit the occurrence of the marine phytoplankton *Thalassiosira pseudonana* in Arctic waters to a greater degree than either factor alone. The results of our experiments and previous studies show that across trophic levels, lower productivity and stress resistance in marine species result from rising water temperatures and trophic mismatches. Since marine hydroids can be considered as a model for macrozoobenthic species, which in turn function as an important food source for higher trophic levels^30^, our results further imply that there will be decreasing productivity at higher trophic levels under the future environmental conditions.

Temperature stress reduced the growth rates (area and polyp count) of *H. echinata* moderately in scenario 1 and significantly in scenario 2 (Fig. 1), while a previous study showed that metabolic rates increase^31^. Rising environmental temperatures generally accelerate biochemical and metabolic reactions in marine ectotherms^32,33^, which can be accompanied by increasing rates of cell damage and reduced growth performance (e.g., in clams, lobsters, and sea cucumbers^34–36^). Eder et al.^31^ found higher cell damage accumulation in juvenile *H. echinata* exposed to 21 °C / low food*,* indicated by higher protein carbonyl contents and most likely induced by increasing production rates of reactive oxygen species. Therefore, in hydroids of scenario 2, with the additional daily temperature step above 21 °C, cell damage most likely occurred at a greater rate, inducing anomalously low growth rates compared to scenario 1. Crucially, mortality rates were considerably higher in the LF/21 °C compared to all other treatments of scenario 1 and 2. Here, the damaging influence of temperature stress seems to outweigh the protective capacities, so that cell and tissue integrity can no longer be maintained. Temperature shifts exceeding 21 °C may thus be critical for some marine ectotherms in terms of growth and vitality^34,35^.

The growth performance of colonies at control temperatures (18 °C) in both scenarios followed the assumptions of the numerical model in most cases, and, therefore, can be explained by relatively simple relationships, such as temperature-driven metabolism and the effect of resource limitation on energy storage and growth. On the other hand, the discrepancies between the experimental data and the model predictions for the highest stress treatments (21 °C/LF) of both scenarios indicate physiological stress reactions that were negligible under control conditions and were therefore not considered in the numerical growth model. For example, the model tended to overestimate the development of the polyps and the area during the high-temperature treatments of scenario 1, whereas it underestimated them in scenario 2. The underestimation of the performance in the most stressed treatment LF/21 °C (scenario 2) can be explained by the fact that mortality could not be implemented in the model. During the experiment, colonies with poor performance died, resulting in higher average numbers of polyps and colony area in the remaining juvenile hydroids. In a similar context, Thomas and colleagues^22^ showed that current ecosystem models overestimate the tolerance of organisms to rising temperatures. They concluded that ectotherms are less tolerant to future warming than to cooling, and that temperatures above the optimum will severely affect both fitness and growth rates of ectotherms and marine phytoplankton^22^.

**Figure 6:**
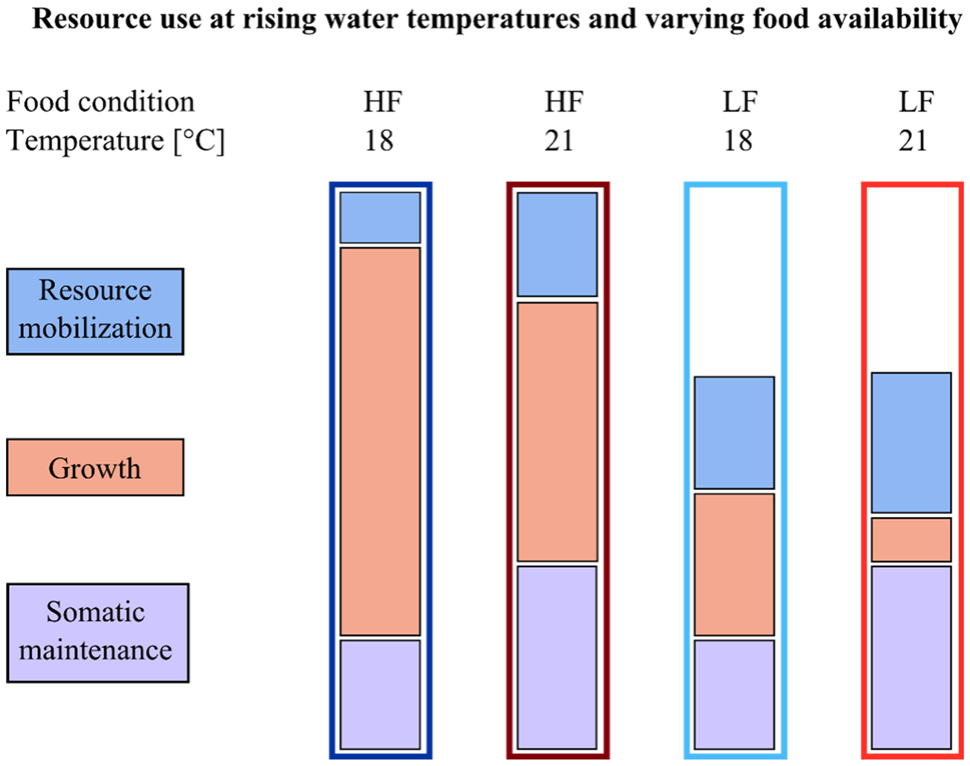
Illustration of resource use in juvenile *H. echinata* at experimental temperature and nutrient conditions in scenario 1 and 2. Low food limits the overall resource availability for the synthesis of biomolecules and energy production necessary for growth (orange boxes) and somatic maintenance (purple boxes), and increases relative costs for resource mobilization (blue boxes) compared to high food. High water temperature (21 °C) increases energetic and nutritive costs for somatic maintenance and intensifies resource mobilization, while energy availability for growth decreases. At low water temperatures (18 °C), energy can be invested in growth and maintenance, but is limited by food intake. Costs for maturation, reproductive buffer, and maturity maintenance have not been taken into account for juvenile hydroids in this figure.

The decreased growth performance at 21 °C compared to 18 °C treatments and the observed differences between model and experimental data can be explained by shifts in energy gain and investment (Fig. 6). The investment of the energy (in the form of ATP) in different physiological functions can vary, depending on the needs of the organism under different environmental conditions^24^. In our study, for example, temperature- and food-stress-induced cell damage accumulation may have forced hydroids to allocate more energy to cell maintenance and survival rather than to area- and polyp growth (Fig. 6). Since physiological processes involved in cell protection, maintenance and repair (e.g., antioxidants capacities, apoptosis and proliferation) could not be included in the numerical growth model, they most likely explain the observed difference between model and experimental data.

We currently lack a mechanistic framework for integrating the effects of multiple interacting physiological functions on the vitality and growth performance of marine invertebrates. The parameterization of ecological growth models is challenging when the experimental/ecological conditions are beyond the optimum of the organisms/ecosystems, and when phenomenological relationships (e.g., temperature-growth relationship) are not sufficient model bases. At the same time, as in our case, they represent an important instrument for drawing attention to physiological stress response mechanisms (e.g., cell protection and repair) that could not be considered in the current model assumptions due to a lack of data, and which should serve as a basis for more complex model approaches in future studies. However, we showed that the growth of juvenile hydroids at temperatures beyond the optimum is a saturation function of energy availability. Temperature effects on growth performances and vitality of *H. echinata* were strongly modulated by food availability. High food availability shifted the thermal optimum of juvenile hydroids to 21 °C as indicated by the growth performance of hydroids at HF/21 °C, which exceeded that of animals at LF/18 °C in both scenarios. The high food treatments covered the energy costs for various physiological functions, such as maintenance and growth of juvenile colonies, despite higher seawater temperatures. Recent studies show a similar trend, with a high diet increasing the acclimatization potential of *Mytilus californianus* and *M. edulis* to temperature- and acidification stress^29,37^. Furthermore, the model by Thomas et al.^22^ for phytoplankton predicts an increase in the minimal requirements for nutrients with rising temperature, and vice versa.

When combining temperature (21 °C) and food stress (LF), hydroid growth rates decreased considerably in both scenarios, and mortality was highest in animals exposed to the most extreme conditions (LF/21 °C, scenario 2), reflecting the mismatch between increasing energy demand and decreasing supply. The combined effect of rising SST and decreased primary and secondary production can be considered to induce energetic imbalances and lower resistances to environmental stress in marine invertebrates such as *H. echinata,* but also in other trophic levels such as that of phytoplankton. The direct relationship between resource availability, allocation, and ecological performance (e.g., growth and reproduction) has been demonstrated for a variety of unicellular and multicellular marine species such as phytoplankton, bivalves, and fish^22,38–41^. However, many studies investigating the interaction between warming and changing nutrient requirements focused on nutrient-rich conditions and showed synergistic effects of eutrophication and warming^42–44^. These interactions may differ strongly in oligotrophic ecosystems, in which temperature-dependent nutrient requirements cannot easily be satisfied. Our current and recent studies on marine and freshwater phytoplankton communities show that the optimum temperature window for growth saturates at a species-specific nutrient concentration, and thus temperatures above the optimum can no longer be compensated for by higher food intake (this study; ^22,45^). Low food availability caused early growth saturation in juvenile hydroids at increasing temperature stress in both scenarios. Similarly, while single experimental treatments of temperature increase or nutrient depletion (oligotrophication) led to increases in resource-use efficiency and phytoplankton community growth, the combined treatment resulted in non-linear responses, reflecting the mismatch between increasing energy demand and decreasing supply^45^. In the present study, excessive maintenance costs (e.g., due to temperature stress) under low nutrition considerably lowered the energy investment into hydroid growth. The high mortality rates of hydroids in the LF/21 °C treatment in scenario 2 indicate severe energy deficiencies, likely inducing cell damage, degradation, and cell and tissue death. While high nutrient supply increases the adaptive potential and the growth performance of marine organisms^28,46^, individuals with resource constraints are exposed to an energy trade-off between survival and growth^29^.

We could show that temperature effects on morphological traits and vitality of marine hydroids were altered significantly by food availability, and that both temperature—and nutrient/nutritional stress should be taken into consideration when predicting future species abundances. Negative effect of food limitation outweighed these of rising water temperatures, while temperature stress responses of *H. echinata* were mitigated by sufficient food supply. We conclude that the interactive effects of ocean warming, increasing energy demand, and decreasing resources may have major impacts on biodiversity and ecosystem function and thus need to be incorporated into environmental management plans. Species living in extreme habitats exposed to highly fluctuating environmental conditions, such as the intertidal, need specific attention and protection arrangements.

## Methods

### Collection of parental colonies

Wildtype colonies of *H. echinata* were collected by staff of the Alfred Wegener Institute within the German Bight around Sylt (55°02′ N; 08°28′ E) with the research vessel MYA II in a depth of 1–3.5 m. Here, the mean annual SST ranges between 1 and 20 °C^47^, and the salinity between 25 and 33 PSU^48^. The sampling took place in April 2016 (scenario 1) and June 2016 (scenario 2). The hydroid colonies were transported to the Carl von Ossietzky University of Oldenburg, and cultured in artificial sea water (Aqua Medic, Germany) at 12 °C and 34 PSU. Before the transport, the hermit crabs were removed from the shells colonized by hydroids. At the University of Oldenburg, the colonies were fed daily with two-day-old living *Artemia salina* nauplii.

### Reproduction and larval settlement

Juvenile hydroids were cultured as described in the Helgoland Manual of Animal Development^27^ Eder et al.^31^. Overall, 10 male and 10 female adult H. echinate colonies were placed together into one big holding tank to release eggs and sperm for fertilization. The fertilized eggs then transformed into larvae. The transformation of the larvae was induced by caesium chloride^49^ following the protocol of as described by Eder et al.^31^, which randomly settled onto black glass tiles (dimensions 10 mm × 10 mm × 2 mm, Mosaikstein, Germany), at least 2–3 larvae on each tile. The colonized glass tiles were then randomly dispersed into the different treatment tanks. As these recruits result from sexual reproduction of overall 20 parental colonies, we can assume high genetic variability of individuals. For individual identification of juvenile colonies, prior to the experiment the glass tiles were engraved underneath with consecutive numbers. After three weeks, the juvenile colonies growing closer to the edges were removed to avoid edge effects, leaving one colony per tile. Prior to the start of the experiments, the colonies were kept in artificial seawater at 18 °C and 34 PSU for 24 h post settlement. From day five of post-settlement onwards, juveniles were fed with two-day-old living *Artemia salina* nauplii.

### Experimental setup

**Figure 7:**
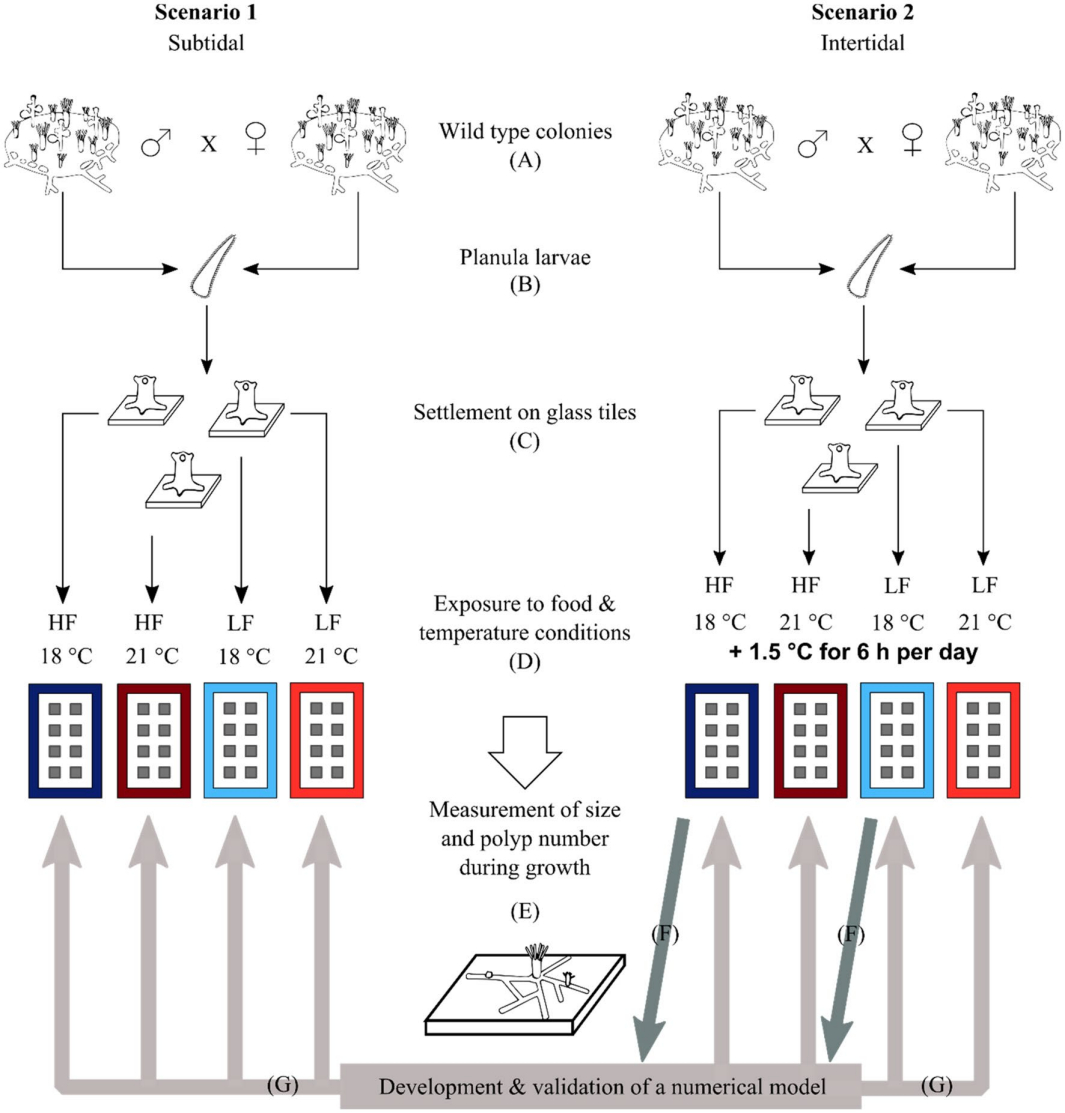
Experimental design for analysing growth performance and mortality of juvenile *H. echinata* in two experiments, scenario 1 (left) and 2 (right). The larvae (**b**) of wild type colonies (**a**) were settled on glass tiles (**c**) and transferred to holding tanks for experimental exposure to variable temperature and food conditions (**d**): HF = high food (dark blue and red tanks); LF = low food (light blue and red tanks); 18 °C = control temperature (blue tanks); 21 °C = high temperature (red tanks). All treatments in scenario 2 contained an extra temperature step of + 1.5 °C for six hours daily. Throughout the experimental timeline and in each treatment, the growth performance of *H. echinata* was monitored (**e**). Additionally, a numerical growth model was developed (**f** dark grey arrows) based on daily analysis of morphological parameters (colony area, polyp number) in hydroids in treatments HF/18 °C and LF/18 °C of scenario 2 and validated (**g** light grey arrows) by comparing simulated and experimental growth data in all treatments of both scenarios.

The influence of temperature and food availability on the growth of *H. echinata* colonies was tested in two different experiments (= scenarios) to evaluate the effect of ambient and future environmental conditions on *H. echinata* in the subtidal (scenario 1, Fig. 7, left panel) and in the intertidal, a habitat characterized by high temperature fluctuations (scenario 2, Fig. 7, right panel). In both scenarios, colonies were exposed to two different temperatures (control and high) cross-factored with two different food conditions (low and high; Fig. 7).

The control temperature of ~ 18 °C simulated the actual sea surface temperature (SST) during summer in the German Bight (data from Helgoland Roads, 2010–2014, http://www.st.nmfs.noaa.gov). The high temperature of ~ 21 °C was chosen according to predicted increasing SST by the end of the century in the North Sea ^8^.

To evaluate the influence of food availability on the growth potential in hydroids as a response to increasing SST, colonies were fed with two-day-old living *A. salina* nauplii (> 1000 nauplii/ml per tank per feeding event) following either a high food (HF) or a low food (LF) scheme (Fig. 7) according to Eder et al.^31^. Colonies with HF were fed five times a week and with LF three times a week. The LF treatments simulated ´food stress` and were patterned on the predicted decrease in primary and secondary production during the next decades^6,50^.

All experimental conditions, including temperature and food, but also salinity, pH, as well as water quality (ammonium, nitrite and nitrate) were constantly monitored. The temperature was measured every ten minutes using HOBO Tidbit v2 Temp Loggers (Onset, USA). The salinity was checked prior to every water exchange (five times a week) with a hand-held refractometer (Arcarda, Germany). Twice a week, the pH was measured with a pH controller (Aqua Medic, Germany), and concentrations of ammonium, nitrite and nitrate were determined with test kits (JBL, Germany). The water quality was checked once a week before the water exchange. If the limit values for ammonium, nitrite and phosphate (0.25 mg/l, 0.2 mg/l and 0.1 mg/l) were exceeded, an additional water change was performed to ensure a consistently good water quality. Juvenile colonies were exposed to a 14 h-light and 10 h-dark cycle according to in-situ conditions in the German Bight in summer (July/August)^31^. Each replicate tank, covered by a lid to reduce evaporation and cooling, was provided with air through an air stone, which was placed in the middle of each tank and connected to a pump (HP-40, Hiblow, Japan). To minimize bacterial and algal growth on the glass tiles they were cleaned once a week, without touching the colonies.

### Scenario 1

**Figure 8:**
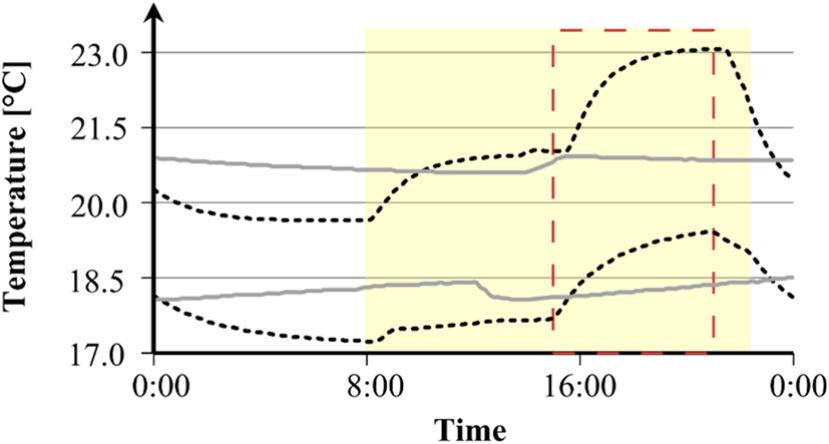
Daily temperature profiles of the control (18 °C; bottom) and high-temperature (21 °C; top) treatments in scenario 1(solid grey lines) and 2 (dotted black lines). In both scenarios, light was provided to experimental animals daily between 8 am and 10 pm (yellow box). All treatments in scenario 2 contained an extra temperature step of + 1.5 °C for six hours daily (between 15:00 and 21:00 h; red box).

In the first experiment (Fig. 7, left panel) conducted in June–August 2016, the growth of 80 *H. echinata* colonies was analysed. The juvenile colonies growing on glass tiles were randomly dispersed into 24 holding tanks containing 100 ml artificial sea water (3–4 glass tiles per tank, 6 replicate tanks per treatment) and exposed for six weeks to, overall, four experimental treatments: HF/18 °C, HF/21 °C, LF/18 °C, LF/21 °C (Fig. 7, left panel). The temperature treatments in this scenario were chosen according to more stable conditions in the subtidal (Fig. 8, grey solid lines), with a control temperature of 18.5 °C ± 0.41 (mean ± SD; Fig. 8, lower grey line) and a high temperature of 20.8 °C ± 0.23 (mean ± SD; Fig. 8, upper grey line). The 24 holding tanks containing the juvenile hydroids were placed in two temperature-constant water baths as described in Eder et al.^31^. A thermostatic heater (Thermo control 300, Eheim, Germany) and two circulation pumps (Voyager Nano, Sicce, Italy) in each water bath kept temperatures constant at 18 °C and 21 °C, respectively.

### Scenario 2

The second scenario (Fig. 7, right panel) was conducted in October-December 2016, testing 71 juvenile *H. echinata* colonies under fluctuating temperature stress*.* The tiles were dispersed randomly into 32 holding tanks (2–3 glass tiles per tank, 8 replicate tanks per treatment) filled with 300 ml artificial sea water and exposed to four different treatments for five weeks, respectively: HF/18 °C + 1.5 °C, HF/21 °C + 1.5 °C, LF/18 °C + 1.5 °C, LF/21 °C + 1.5 °C (Fig. 7, right panel). This scenario contained an additional temperature step (+ 1.5 °C) for all treatments, to implement daily temperature fluctuations and mimic natural variations in the intertidal during high and low tide^51^; Fig. 8, black dotted lines). Therefore, the control temperature treatments of 18.2 °C ± 0.60 was increased daily for six hours to 19.5 °C ± 0.17 (mean ± SD; Fig. 8, lower black line), and the high temperature treatments of 20.7 °C ± 0.55 to 22.3 °C ± 0.06 (mean ± SD; Fig. 8, upper black line). The holding tanks were placed into temperature-controlled incubators (MIR-554, Panasonic Healthcare Co., Japan & MIR-553, Sanyo Electric Co., Japan) for each temperature regime.

### Growth rates and mortality

Throughout the experiment, the colonies developed normally without any signs of polyp or tentacle deformation. Each colony was morphometrically analyzed on a weekly basis in terms of colony area and number of polyps, as indicators for individual growth performance. These parameters were determined throughout both experiments by analyzing weekly photographs of colonies (Fig. 1), taken through a binocular microscope (Leica M205 C, Leica Microsystems, Germany) between the ages of 5–46 days post-settlement (scenario 1) and 8–36 days post-settlement (scenario 2). In scenario 1, the photographs of animals in the HF and LF treatments were taken three days apart for logistical reasons. The area of each colony was determined graphically by an automated script developed using Matlab (Version R2015b, The MathWorks, Inc., USA). The script identifies the shape of the largest patch on each glass tile and excludes the spaces between the stolonal channels. Geometric patterns were not taken into consideration to counteract potential morphological differences based on genetic variations or similarities (e.g. sheet or runner like colonies). For the determination of polyp number, only completely developed polyps equipped with tentacles were counted, whereas buddies were ignored. The juveniles did not reach sexual maturity during the experiment, therefore the colonies consisted exclusively of feeding polyps.

Additionally, we analyzed mortality rates by day 35 post-settlement for both scenarios, which were characterized by visible colony-wide signs of cell necrosis and tissue detachment from the surface.

In the treatments HF/18 °C and LF/18 °C of the second scenario, daily pictures of eight colonies (four colonies of each treatment) were taken between day 8 and 27 post-settlement to develop and validate a numerical growth model.

### Model development and validation

To identify physiological response mechanisms of juvenile hydroids exposed to nutrient and temperature stress, we developed a numerical growth model based on morphological data. The effect of environmental stress was simulated by phenomenological relationships, such as temperature-driven metabolism and the negative effects of resource limitation on growth rates. To identify unexpected trends and features in the experimental growth data, the results of the model simulations were compared to the experimental data.

The model simulated the day-to-day growth of the colonies through building up feeding polyps and the stolon system. The polyps were described by nodes of a growing network connected by stolon branches. The energy was treated as an artificial, dimensionless quantity that was distributed over the nodes of the network. Food uptake resulted in an increase of the energy amount of every feeding polyp. Energy loss was accounted for by temperature-dependent rest and activity respiration according to van’t Hoff’s rule and the costs for stolon and polyp growth. The energy of a node was equally distributed to adjacent stolons and polyps at a fixed distribution rate, whereby pressure inequalities in stolon branches were not considered due to an assumed constant stolon diameter.

The model was initialized by one feeding polyp. Depending on the available energy, the colony developed feeding polyps first to increase its energy intake. Then, if enough energy was left, the colony built up stolon branches of a certain length in an arbitrary direction. The energy needed for stolon growth was proportional to the length and had to exceed the parameterized amount for the growth of a stolon of reference length. The minimum distance between two polyps was also parameterized, as well as the energy needed for this process.

Parameter values were partly taken from the experiments and partly estimated by automatic parameter optimization (Supplementary Table 1). For this, the parameters of the model were trained to the lab data of the LF/18 °C treatment (scenario 2) in respect to the number of feeding polyps and area of the colony. The trained parameter set was then used for all other simulations. The simulation was repeated 100 times per treatment, with the respective temperature and food scheme and randomized stolon growth. The model was programmed in C and ran for 43 days (scenario 1) and 37 days (scenario 2) with a time step of 6 h to simulate the periods of frequent temperature stress in scenario 2 (= additional temperature step). The model was calibrated using the treatment LF/18 °C (scenario 2).

### Statistics

We compared the growth performance over time between experimental data and simulated data, for both scenarios separately. Area and polyp growth rates were compared by a pairwise Wilcoxon test with multiple-testing adjustment (Bonferroni-Holmes) in R (R version 3.5.1, R Core Team 2018). The 18 °C high-food condition (HF/18 °C) was set as the reference group. The respective parameters ($\alpha ,b$) were calculated as follows:

1. Area growth (colony area as a function of time) of the individual colonies was square root transformed and fitted with a linear model of the form, $$\sqrt[4]{{\varvec{A}}}\left( {\varvec{t}} \right) = {\varvec{a}} + {\varvec{bt}} + {\varvec{\varepsilon}}\left( {\varvec{t}} \right),\user2{ }$$$$[4]$$$${{\varvec{A}}}\left( {\varvec{t}} \right) = {\varvec{a}} + {\varvec{bt}} + {\varvec{\varepsilon}}\left( {\varvec{t}} \right),\user2{ }$$ where $${\varvec{A}}$$ is the colony area, $${\varvec{a}},{\varvec{b}}$$ represent intercept and slope of the fitted area growth curve, respectively, and $${\varvec{\varepsilon}}$$ is the model error.
2. The polyp growth (number of polyps as a function of time) was fitted using the parametric Gompertz function without transformation, usually used to describe tumour growth kinetics (Laird, 1964) as follows: ${\varvec{N}}\left( {{\varvec{t}};{\varvec{\alpha}},{\varvec{\beta}}} \right) = {\varvec{e}}^{{{\varvec{\alpha}}/{\varvec{\beta}}\left( {1 - {\varvec{e}}^{{ - \user2{\beta t}}} } \right)}} ,\user2{ }$ where ${\varvec{\alpha}} > 0$ is the initial growth constant, $\mathop {\lim }\limits_{{{\varvec{t}} \to 0}} \frac{{\varvec{d}}}{{{\varvec{dt}}}}{\varvec{N}}\left( {\varvec{t}} \right) = \user2{\alpha N}\left( {\varvec{t}} \right),\user2{ }$ i.e. initial exponential growth, $\mathop {\lim }\limits_{{{\varvec{t}} \to 0}} {\varvec{N}}\left( {\varvec{t}} \right) = \exp \left( {\user2{\alpha t}} \right)$, and ${\varvec{\beta}} > 0$ is the growth constant at the maximum growth rate, i.e., $\frac{{\varvec{d}}}{{{\varvec{dt}}}}{\varvec{N}}\left( {{\varvec{t}} = {\varvec{t}}_{{\mathbf{i}}} } \right) = \user2{\beta N}\left( {{\varvec{t}} = {\varvec{t}}_{{\mathbf{i}}} } \right) = \frac{{\varvec{\beta}}}{{\varvec{e}}}{\varvec{e}}^{{{\varvec{\alpha}}/{\varvec{\beta}}}}$, with ${\varvec{t}}_{{\mathbf{i}}} = \left( {\ln \frac{{\varvec{\alpha}}}{{\varvec{\beta}}}} \right)/{\varvec{\beta}}$ defining the inflexion point of the (sigmoidal) growth curve. The function follows the trend of a logistic growth curve, but is characterized by asymmetric growth through saturation. In both experiments, saturation was probably not reached, but in scenario 2, growth declined towards the end of the experiment. This decline was set as an indicator for the end of the optimal growth phase and, therefore, the end of the experiment. The ${\varvec{\beta}}$ parameter determined for the experimental polyp growth curve of each colony was always significantly lower than the respective ${\varvec{\alpha}}$ parameter and did not differ significantly across the conditions. This allowed us to compare the conditions in terms of the ${\varvec{\alpha}}$ parameter only.

The analysis of numerical growth model data followed the same procedure. Then, the growth curves over time (polyp number, colony area) for the experimental data and the simulation were qualitatively compared based on the shape and the discrepancies between the curves. Parametric functions to experimental growth curves were fitted using NonlinearModelFit in Wolfram Mathematica (Version 11, Wolfram Research, UK). R was used for statistical analysis and for producing graphical output. The effect sizes (Cohen’s effect size, Odds Ratio) are presented in the supplements (Supplementary Table 4, 5).

In addition, we analyzed the experimental data in terms of mortality, which was defined as the proportion of colonies that died 35 days after settlement, using the proportion test in R.

### Animal rights

All applicable international, national, and/or institutional guidelines of the University Oldenburg and the federal state Lower Saxony (Germany) for the care and use of animals were followed.

## Supplementary Information

Supplementary Information

## Data Availability

All data used for the growth analysis of scenario 1 and scenario 2 are available at 10.5063/DZ06QQ (Knowledge Network for Biocomplexity).
