# Author Correction: Diminished growth and vitality in juvenile *Hydractinia echinata* under anticipated future temperature and variable nutrient conditions

**DOI:** 10.1038/s41598-021-94493-x

**Published:** 2021-08-02

**Authors:** Daniel Tschink, Gabriele Gerlach, Michael Winklhofer, Cora Kohlmeier, Bernd Blasius, Laura Eickelmann, Yvonne Schadewell, Julia Strahl

**Affiliations:** 1 Institute of Biology and Environmental Sciences, Carl Von Ossietzky University Oldenburg, Carl von Ossietzky Str. 9-11, 26111 Oldenburg, Germany; 2 Institute for Chemistry and Biology of the Marine Environment (ICBM), Carl Von Ossietzky University Oldenburg, Carl von Ossietzky Str. 9-11, 26111 Oldenburg, Germany; 3 Helmholtz Institute for Functional Marine Biodiversity at the University of Oldenburg (HIFMB), Ammerländer Heerstr. 231, 26129 Oldenburg, Germany; 4 Centre of Excellence for Coral Reef Studies and School of Marine and Tropical Biology, James Cook University, Townsville, QLD 4811 Australia; 5 Alfred Wegener Institute Helmholtz Centre for Polar and Marine Research, Am Handelshafen 12, 27570 Bremerhaven, Germany

Correction to: *Scientific Reports* 10.1038/s41598-021-86918-4, published online 05 April 2021

The original version of this Article contained an error in the Data Availability section, where

“The morphological (biomass), physiological (standard metabolic rate), and biochemical (contents of protein and protein carbonyl) data for scenario 1, including supplementary information, are available online at 10.1016/j.jembe.2018.07.009. All data used for the growth analysis of scenario 2 are shown in the manuscript and will be available online before publishing.”

now reads:

“All data used for the growth analysis of scenario 1 and scenario 2 are available at 10.5063/DZ06QQ (Knowledge Network for Biocomplexity).”

In addition, the Supplementary Information file contained tracked changes.

The original Article and accompanying Supplementary Information file have been corrected.

