# Supplementary material for: Diminished growth and vitality in juvenile Hydractinia echinata under anticipated future temperature and variable nutrient conditions
Source: Sci Rep. 2021 Apr 5;11:7483. doi: 10.1038/s41598-021-86918-4 (PMC8021570; doi:10.1038/s41598-021-86918-4)
Supplement: Supplementary file 1 — Supplementary Information [file 41598_2021_86918_MOESM1_ESM.docx]

**Supplementary material to**

**Diminished growth and vitality in juvenile *Hydractinia echinata* under anticipated future temperature and variable nutrient conditions**

**Authors:**

Daniel Tschink^1*^ (ORCID: 0000-0001-8683-2534),

Gabriele Gerlach^1,3,4^ (ORCID: 0000-0001-5246-944X),

Michael Winklhofer^1^ (ORCID: 0000-0003-1352-9723),

Cora Kohlmeier^2^,

Bernd Blasius^2,3^ (ORCID: 0000-0002-6558-1462),

Laura Eickelmann^1,2^ (ORCID: 0000-0001-6707-3149),

Yvonne Schadewell^1,2^ (ORCID: 0000-0001-8969-7972),

Julia Strahl^1,3,5^ (ORCID: 0000-0001-9496-4019)

*^1^Institute of Biology and Environmental Sciences, Carl von Ossietzky University Oldenburg, Carl von Ossietzky Str. 9-11, 26111 Oldenburg, Germany*

*^2^Institute for Chemistry and Biology of the Marine Environment (ICBM), Carl von Ossietzky University Oldenburg, Carl von Ossietzky Str. 9-11, 26111 Oldenburg, Germany*

*^3^Helmholtz Institute for Functional Marine Biodiversity at the* *University of Oldenburg (HIFMB), Ammerländer Heerstr. 231, 26129 Oldenburg, Germany*

*^4^Centre of Excellence for Coral Reef Studies and School of Marine and Tropical Biology, James Cook University, Townsville, Queensland, 4811 Australia*

*^5^Alfred Wegener Institute Helmholtz Centre for Polar and Marine Research, Am Handelshafen 12, 27570 Bremerhaven, Germany*

**Corresponding author:*

*Daniel.Tschink@uol.de / Phone:* +49 441 798 3576 */ Institute of Biology and Environmental Sciences, Carl von Ossietzky University Oldenburg, Carl von Ossietzky Str. 9-11, 26111 Oldenburg, Germany*

**Keywords**

Cnidaria, growth rate, temperature fluctuation, resource limitation, growth model, temperature-nutrition interplay

***Supplementary Table 1*** **Parameters used for modelling the growth of juvenile *H. echinata*** in a numerical growth model. The first three values (dia_polyp, ref_dia_stolon, max_links) are estimates from the lab experiments, e_food is set to an arbitrary value, the remaining values are rounded values from automatic parameter optimization by the low food 18°C treatment and verified at high food 18°C treatment (scenario 1).

| **Name** | **Value** | **Unit** | **Meaning** |
| --- | --- | --- | --- |
| *dia_polyp* | 0.03 | mm | estimated polyp diameter |
| *ref_dia_stolon* | 0.007 | mm | reference stolon diameter |
| *max_links* | 5.00 | - | maximum number of links per node |
| *e_food* | 1.00 | - | energy increase of nutritive polyp per feeding |
| *e_polyp* | 0.10 | - | energy needed for new polyp |
| *e_stolon* | 1.36 | - | energy needed for new stolon with  reference length |
| *r_rest* | 0.001 | d^-1^ | rest respiration rate at 10°C |
| *r_act* | 0.06 | d^-1^ | activity respiration rate at 10°C |
| *ref_len* | 0.93 | mm | reference length of stolon growth |
| *D* | 0.26 | d^-1^ | energy distribution rate |
| *initial_energy* | 0.22 | - | energy of the first polyp on day 1 |
| *min_age* | 0.58 | d | minimum age of node for polyp conversion |

| *Supplementary Table 2* Size development of juvenile *H. echinata* during the first weeks of development in scenario 1 and 2. Median, mean, standard deviation (SD) and replicate number (N) over time (growth rate) and at age 34-36 days post-settlement (final) at varying food (high, low) and temperature (18°C, 21°C) conditions.  Colony area | | | | | | | | | |
| --- | --- | --- | --- | --- | --- | --- | --- | --- | --- |
|  | | | Growth rate | | | | Final | | |
| Scenario | Treatment | | N | Median  [mm^1/2^/day] | Mean  [mm^1/2^/day] | SD  [mm^1/2^/day] | Median [mm^2^] | Mean  [mm^2^] | SD [mm^2^] |
|  | Food | Temperature  [°C] |  |  |  |  |  |  |  |
| 1 | High | 18 | 18 | 0.045 | 0.045 | 0.009 | 10.0 | 12.0 | 6.7 |
|  |  | 21 | 20 | 0.038 | 0.041 | 0.010 | 6.7 | 8.1 | 5.9 |
|  | Low | 18 | 19 | 0.032 | 0.034 | 0.008 | 5.0 | 6.7 | 5.0 |
|  |  | 21 | 20 | 0.025 | 0.026 | 0.012 | 2.3 | 4.4 | 5.0 |
| 2 | High | 18 | 11 | 0.037 | 0.034 | 0.015 | 6.3 | 8.4 | 6.1 |
|  |  | 21 | 9 | 0.037 | 0.037 | 0.005 | 7.1 | 8.5 | 3.3 |
|  | Low | 18 | 13 | 0.030 | 0.030 | 0.008 | 3.4 | 4.3 | 2.9 |
|  |  | 21 | 7 | 0.035 | 0.033 | 0.008 | 5.5 | 5.5 | 2.0 |

| *Supplementary Table 3* Polyp development of juvenile *H. echinata* during the first weeks of development in scenario 1 and 2. Median, mean, standard deviation (SD) and replicate number (N) over time (growth rate) and at age 34-36 days post-settlement (final) at varying food (high, low) and temperature (18°C, 21°C) conditions.  Polyps per colony | | | | | | | | | |
| --- | --- | --- | --- | --- | --- | --- | --- | --- | --- |
|  | | | Growth rate | | | | Final | | |
| Scenario | Treatment | | N | Median  [1/day] | Mean  [1/day] | SD  [1/day] | Median | Mean | SD |
|  | Food | Temperature  [°C] |  |  |  |  |  |  |  |
| 1 | High | 18 | 13 | 0.120 | 0.132 | 0.033 | 44.0 | 56.1 | 28.9 |
|  |  | 21 | 13 | 0.120 | 0.120 | 0.029 | 33.5 | 34.9 | 24.8 |
|  | Low | 18 | 12 | 0.108 | 0.113 | 0.023 | 30.0 | 31.0 | 17.0 |
|  |  | 21 | 13 | 0.093 | 0.095 | 0.027 | 12.5 | 14.7 | 10.4 |
| 2 | High | 18 | 10 | 0.095 | 0.090 | 0.017 | 30.0 | 27.8 | 15.2 |
|  |  | 21 | 5 | 0.091 | 0.089 | 0.007 | 20.0 | 23.7 | 13.0 |
|  | Low | 18 | 8 | 0.083 | 0.085 | 0.021 | 13.0 | 17.5 | 13.3 |
|  |  | 21 | 2 | 0.091 | 0.091 | 0.006 | 15.5 | 16.3 | 7.7 |

***Supplementary Table 4***  **Cohen’s effect size *d* with Hedges & Olkin correction** for area growth rate in scenario 1 and 2. As opposed to the p-values, the effect sizes are not adjusted for multiple comparisons. An effect size of |*d*| < 0.3 is considered biologically irrelevant. Strong effects are characterized by an effect size |*d*| > 0.8. The number *n* of growth curves that allowed for a robust fit is given in parenthesis for each condition.

| **Cohen’s effect size *d*** | | | | | | | | |
| --- | --- | --- | --- | --- | --- | --- | --- | --- |
| Treatment | Area | | | Polyps | | | | |
|  | HF/18 °C | HF/21 °C | LF/18 °C | HF/18 °C | HF/21 °C | | LF/18 °C | |
| **Scenario 1** | | | | | | | | |
| HF/21 °C | -0.41 |  |  | -0.38 | |  | |  |
| LF/18 °C | -1.41 | -0.95 |  | -0.65 | | -0.26 | |  |
| LF/21 °C | -2.16 | -1.73 | -0.89 | -1.18 | | -0.84 | | -0.66 |
| **Scenario 2** | | | | | | | | |
| HF/21 °C | -0.11 |  |  | -0.05 | |  | |  |
| LF/18 °C | -0.58 | -0.71 |  | -0.23 | | -0.2 | |  |
| LF/21 °C | -0.48 | -0.64 | 0.07 | 0.07 | | -0.24 | | 0.26 |

***Supplementary Table 5***  **Odds Ratio for mortality in LF21** condition relative to other conditions in scenario 2. The fraction in parentheses indicates the number of colonies that died within 35 days after settlement relative to the initial number of colonies.

| **Odds Ratio** | | | | |
| --- | --- | --- | --- | --- |
| Treatment |  | HF18 | HF21 | LF18 |
|  | Colonies died / initial number | 15 / 111 | 8 / 106 | 12 / 110 |
| LF21 | 28 / 109 | 2.2 | 4.23 | 2.82 |
